# A design of experiments approach for the rapid formulation of a chemically defined medium for metabolic profiling of industrially important microbes

**DOI:** 10.1371/journal.pone.0218208

**Published:** 2019-06-12

**Authors:** Chloe Singleton, James Gilman, Jessica Rollit, Kun Zhang, David A. Parker, John Love

**Affiliations:** 1 The Exeter Microbial Biofuels Group, College of Life and Environmental Sciences, The University of Exeter, Exeter, Devon, United Kingdom; 2 Department of Life Sciences, Imperial College London, London, United Kingdom; 3 Shell Technology Centre, Houston, Texas, United States of America; USDA-Agricultural Research Service, UNITED STATES

## Abstract

*Geobacillus thermoglucosidans* DSM2542 is an industrially important microbe, however the complex nutritional requirements of *Geobacilli* confound metabolic engineering efforts. Previous studies have utilised semi-defined media recipes that contain complex, undefined, biologically derived nutrients which have unknown ingredients that cannot be quantified during metabolic profiling. This study used design of experiments to investigate how individual nutrients and interactions between these nutrients contribute to growth. A mathematically derived defined medium has been formulated that has been shown to robustly support growth of *G*. *thermoglucosidans* in two different environmental conditions (96-well plate and shake flask) and with a variety of lignocellulose-based carbohydrates. This enabled the catabolism of industrially relevant carbohydrates to be investigated.

## Introduction

The *Geobacillus* [1] have great potential for use as an industrially relevant microbial chassis [2–4]. In particular, *Geobacillus thermoglucosidans* DSM 2542 (NCIMB 1195) (hereafter *G*. *thermoglucosidans*) is a promising candidate as it has previously been engineered for bioethanol production [5].

Some *Geobacillus* species, including *G*. *thermoglucosidans*, can utilise lignocellulose based carbohydrates for growth, including hexoses, pentoses, di- and oligo-saccharides [6]. However, many of these experiments were performed in media that included biologically derived nutrients (bionutrients) such as tryptone, yeast extract and casamino acids, that are typically considered constituents of complex media and may support bacterial growth [5,7–11] (S1 Table). Carbohydrate utilization by *G*. *thermoglucosidans* in these studies was therefore determined against a background of ‘unknown’ nutrients which may influence the catabolism of these carbohydrate substrates. Attempts have been made to design defined media recipes that do not require bionutrient additives, [12–16] but these were for alternative, thermophilic species (*Bacillus stearothermophilus* LLD-R, associated mutant strains and *Bacillus stearothermophilus* 1503) that are likely to have different nutritional requirements to that of *G*. *thermoglucosidans*. Typically, media are developed based on previous recipes and utilise the traditional method of varying one-factor-at-a-time (OFAT) where all but one ingredient (factor) is fixed [12,13]. Although this can lead to determination of how individual factors influence growth of cultures, such as particular amino acids or vitamins, this method does not consider the effect of potential interactions between factor.

Design of Experiments (DoE) uses a multifactorial approach employing statistical methodologies to both design and analyse an experimental process. In a ‘designed’ experiment a number of factors are simultaneously varied, such as phosphate, vitamin and amino acid concentrations, and the resultant impact on the system response, such as bacterial growth, is measured. As well as simultaneous factor variation (Factorial Design), DoE is underpinned by three other important principles to allow for robust, valid experimentation [17]: at least some experiments, known as treatments, must be replicated; treatments must be randomized to protect against hidden factors; and finally treatments must be performed in blocks to control for technical sources of variation. The statistical approach used to design an experimental process, allows conformity to all four principles and complete exploration of large experimental design spaces whilst minimising the number of experiments and resource requirements. In addition, DoE software such as JMP Pro (SAS Institute Inc. USA), enables visualization of complex interactions between multiple factors and provides predictions of the biological system or process. The use of DoE for fermentation medium optimisation for a variety of microorganisms was discussed in a review paper by Singh et al in 2017 [18]. More relevantly for *Geobacillus*, statistically based experimental designs have been used to develop media for improved production of industrially important thermostable enzymes including: lipase production [19], avicelase production [20] and amidase production [21]. Previously, work done in this laboratory used DoE methodology to describe metabolic interactions in genetically engineered yeast [22].

The aim of this study was to investigate the complex carbohydrate catabolism of *G*. *thermoglucosidans*. However, growth of *G*. *thermoglucosidans* cultures in the recommended defined Ammonium Sulphate Medium (Zuvasyntha), was inconsistent without supplementation with 1 g L^-1^ yeast extract. We therefore used a DoE approach to mathematically formulate a truly ‘defined’ media recipe for robust growth of *G*. *thermoglucosidans*. In addition, the experimental process demonstrated some counterintuitive results which could only be discovered by this framework.

## Materials and methods

### *Geobacillus thermoglucosidans* strain and growth media

The type strain of *Geobacillus thermoglucosidans* (DSM2542) was obtained from the DSMZ (Brunswick, Germany). Cultures were freeze-dried ampoules and rehydrated as required following the DSMZ standard protocol. Complex media reagents were purchased from Becton Dickson (Berkshire, United Kingdom). All other reagents were purchased from either Fisher Scientific (Leicestershire, United Kingdom) or Sigma-Aldrich (Dorset, United Kingdom). For all solid media, agar was supplemented to 15 g L^-1^. Modified Luria-Bertani broth (mLB) was used to analyse growth of *G*. *thermoglucosidans* cultures in complex media. mLB comprised: 10 g L^-1^ Tryptone, 5 g L^-1^ yeast extract, 5 g L^-1^ NaCl, 1.05 mM C_6_H_9_NO_6_, 0.91 mM CaCl_2_, 0.59 mM MgSO_4_ and 0.04 mM FeSO_4_ [23]. *G*. *thermoglucosidans* cultures were also grown in the defined Ammonium Sulphate Medium (ASM). ASM comprised: 10 g L^-1^ of either glucose or xylose, 50 mM MOPS pH 7, 25 mM (NH_4_)_2_SO_4_, 20 mM NaH_2_PO_4_, 10 mM K_2_SO_4_, 8 mM citric acid, 5 mM MgSO_4_, 1.65 mM Na_2_MoO_4_, 0.08 mM CaCl_2_, trace metal solution (600 μM H_2_SO_4_, 200 μM FeSO_4_, 100 μM MnSO_4_, 50.1 μM ZnSO_4_, 22.5 μM NiSO_4_, 20 μM CoSO_4_, 13 μM H_3_CO_3_ and 10 μM CuSO_4_). The media was supplemented with either 1 g L^-1^ yeast extract or a vitamin solution (951 μM glutamic acid, 425 μM serine, 336 μM threonine, 11.9 μM biotin and 11.9 μM thiamine hydrochloride). Finally, *G*. *thermoglucosidans* cultures were grown in 10 g L^-1^ yeast extract added to deionised water.

### Characterisation of culture growth

Growth of *G*. *thermoglucosidans* cultures was monitored by the change in optical density, from time 0 h, at a wavelength of 600 nm (Δ_OD600_). As inoculation with single colonies never resulted in bacterial growth, starter cultures were prepared by collecting approximately 10 μl *G*. *thermoglucosidans* from a confluent plate culture that had been incubated overnight at 55 °C and resuspending in 1 ml of 5 g L^-1^ yeast extract solution. The resulting bacterial suspensions were used to inoculate aliquots of the appropriate media to an optical density at 600 nm (OD600) of 0.04 (typically a 1 in 200 dilution). In the case of samples grown in flask format, 50 ml of the appropriate media was inoculated with starter culture in 250 ml Erlenmeyer flasks. Flasks were incubated at 60 °C, with shaking at 220 rpm. In the case of samples grown in 96-well microplate format, 1 ml aliquots of the appropriate media were inoculated from the starter cultures. 200 μl aliquots of the resulting bacterial suspensions were loaded onto 96-well plates using a Corbett Robotics CAS-1200 (Qiagen, Netherlands). Microplates were incubated using PHMP thermoshakers (Grant Instruments, United Kingdom). Incubation was at 60 °C, with shaking at 800 rpm. To minimise the effect of position dependant bias [24], sample aliquots were loaded in a Latin rectangle design; technical replicates of each starter culture were not represented more than once on any given row or column. As microplates with lid covers have been shown to suffer from significant loss of culture in the outermost wells through evaporation [25], wells at the microplate periphery were filled with sterile deionised water instead of bacterial culture. In all instances, culture absorbance was measured using a Tecan Infinite 200 PRO microplate reader (Tecan, Switzerland). For cultures grown in flask format, 200 μl sample aliquots were loaded into 96-well plates for analysis at the relevant time points.

### Determining carbohydrate utilization

Bacterial cultures were grown in 250 ml flasks and at the relevant time points, 200 μl sample aliquots were clarified at 10,000 rpm for 5 min. 100 μl of the resulting supernatant was subsequently added to 900 μl of 10 mM H_2_SO_4_. Carbohydrate content was analysed using an Aglient (California, USA) 1260 Infinity HPLC system with a Rezex monosaccharide H^+^ column (Phenomenex, California, USA), equilibrated with 0.005% H_2_SO_4_ mobile phase at 50 °C and 0.6 ml min^-1^. 40 μl of sample was injected and carbohydrates were eluted from the column using an isocratic elution over 30 min. Carbohydrates were detected by a refractive index (RI) detector with an initial temperature of 40 °C, RI detector range of 500 μRIU/V and recorder range of 512.00 μRIU.

### Design of experiments

Design of experiments (DoE) and statistical modelling for the development of a defined *G*. *thermoglucosidans* growth medium was performed using JMP pro software version 12 (SAS institute, North Carolina, USA). Defined media recipes for a variety of microorganisms were used to generate a list of commonly used ingredients (S2 Table). The list included nine mineral salts which included combinations of ammonium, potassium and sodium cations; and carbonate, chloride, nitrate and sulphate anions. Citric acid and urea were identified as potential additional sources of carbon and nitrogen, respectively. A MOPS buffer was included, as regulation of media pH has been shown to be essential for maintaining *Geobacillus* growth [26]. EDTA was included as a chelating agent [13,27]. The four trace elements found in mLB, calcium chloride, iron sulphate, magnesium sulphate and nitrilotriacetic acid (NTA), [23] were included in the experimental design, as were commercially available amino acid (50 x MEM amino acids solution and 100 x MEM non-essential amino acids solution, both ThermoFisher Scientific, United Kingdom) and vitamin solutions (MEM vitamin solution, ThermoFisher Scientific, United Kingdom, supplemented with 1.19 mM biotin). Commercial amino acid and vitamin mixes were used to ensure that no single essential micronutrient was likely to become limiting. In addition, a trace metal solution was developed based on the reviewed defined media recipes (S3 Table). Finally, yeast extract, at a maximum concentration of 1 g L^-1^ was included to ensure that growth would be observed during the early iterations. In total, 21 continuous factors were defined as minimal media ingredients. For the first iteration a Fractional Factorial Screening Design was chosen. Screening Designs often involve a large number of factors and allow for initial differentiation of significant and non-significant factors as well as an estimation of the magnitude of the significant factors. A Full Factorial design, including 21 factors, would require almost 17 million experimental treatments, therefore the Fractional Factorial platform of the JMP software was used to generate 64 experimental treatments, randomly distributed into eight blocks (S4 Table). Each block was comprised of eight treatments, and to provide information on technical error, a positive control and a negative control. The design provided an estimate for all 21 main effects, as well as a limited number of randomly selected first order interactions.

The second iteration comprised nine continuous factors, the media ingredients that were identified in the first iteration as influencing *G*. *thermoglucosidans* growth. The Custom Design platform of the JMP software was used to construct a design that balanced the need to maximise the information that could be gathered from the experiment whilst minimising resources and time. A 56-treatment experimental design was generated that provided estimates for all the main effects as well as estimates for all possible first order interactions between the ingredients. The 56 treatments were randomly distributed into seven blocks (S5 Table). In both iterations, each experimental block was analysed on an individual 96-well microplate. Two technical replicates of each treatment were loaded per 96-well plate in a Latin rectangle design, and three biological replicates of each block were performed, from independent starter cultures. In all treatments, glucose was added at a final concentration of 10 g L^-1^. To account for any potential batch effects, positive controls, *G*. *thermoglucosidans* grown in 10 g L^-1^ yeast extract solution, were included on each microplate.

### Partial least squares modelling

Partial least squares (PLS) modelling was used to describe the relationship between media ingredients, their first-order interactions (*X*) and culture Δ_OD600_ (*Y*). PLS infers the relationship between matrices of predictor (*X*) and response (*Y*) variables using the assumption that the covariance of the two matrices can be accurately inferred through a smaller number of underlying, or latent, variables (LVs) which are not directly observed or measured [28]. In all instances, the non-iterative PLS algorithm (NIPALS) was applied. The optimum number of LVs to extract from the data must be carefully considered, as models containing large numbers of LVs risk being overfit to the training data, and can thus be inadequately general [29]. Therefore, KFold cross validation with K = 7 was used to optimise the number of extracted LVs. For both DoE iterations, 15 PLS models were fit that extracted between one and 15 LVs from the original data. The prediction error (Root Mean Predicted Residual Error Sum of Squares) for each of these models was calculated, and the optimum model was judged to be the one with the smallest number of LVs whose Root Mean PRESS was not statistically significantly greater than the model with the lowest error [30]. Significance was determined using the van der Voet T^2^ test [31]. The PLS algorithm calculates a summary statistic, termed the Variable Importance in Projection (VIP) score [32,33] that can be used to determine the contribution of a given predictor variable to model output (*e*.*g*. the importance of a given media ingredient or an interaction between ingredients in determining culture Δ_OD600_). The higher the VIP score of a given media component, the larger the predicted effect of that component on culture OD600. A threshold value of 0.8 is commonly accepted, above which predictor variables are judged to have a significant impact on the model output [34]. Conversely, *x* variables with low VIP scores are candidates for removal from subsequent design iterations. The VIP score can be interpreted in combination with the model coefficients returned by a given predictor variable to determine the magnitude of the contribution to the measured output. In addition, the model coefficients indicate if this contribution is positive or negative [22].

### Stepwise regression and artificial neural network modelling

The results of the second DoE iteration were also modelled using Stepwise Regression. All possible linear regression models were fit, allowing a maximum of six terms per model with Heredity Restriction. The goodness-of-fit of the resulting models was assessed using the second order Akaike Information Criterion (AICc), calculated as:

AICc=-2(logLikelihood)+2k+[2k(k+1)÷(n-k-1)]

where *n* is the number of observations used in the model, *k* is the number of parameters estimated in the model and *ogLikelihood* is the natural logarithm of the likelihood function. For each candidate model (*i*), the Kullback-Leibler distance from the optimum obtained model (*i*.*e*. the model with the smallest AICc, AICc_min_) was calculated as:

$$\Delta i={AICc}_{i}-{AICc}_{min}$$

An artificial neural network (ANN) was subsequently used to create a weighted ensemble of those regression models for which Δ_*i*_ was less than 2.0 [35]. The ANN consisted of a single hidden layer that contained three nodes. Sigmoid activation functions were used. Holdback cross validation was used for ANN construction; 19 of the media formulations that were defined in the second DoE iteration were randomly selected and withheld from ANN training to serve as a validation set.

## Results

### Growth of *Geobacillus thermoglucosidans*

**Fig 1:**
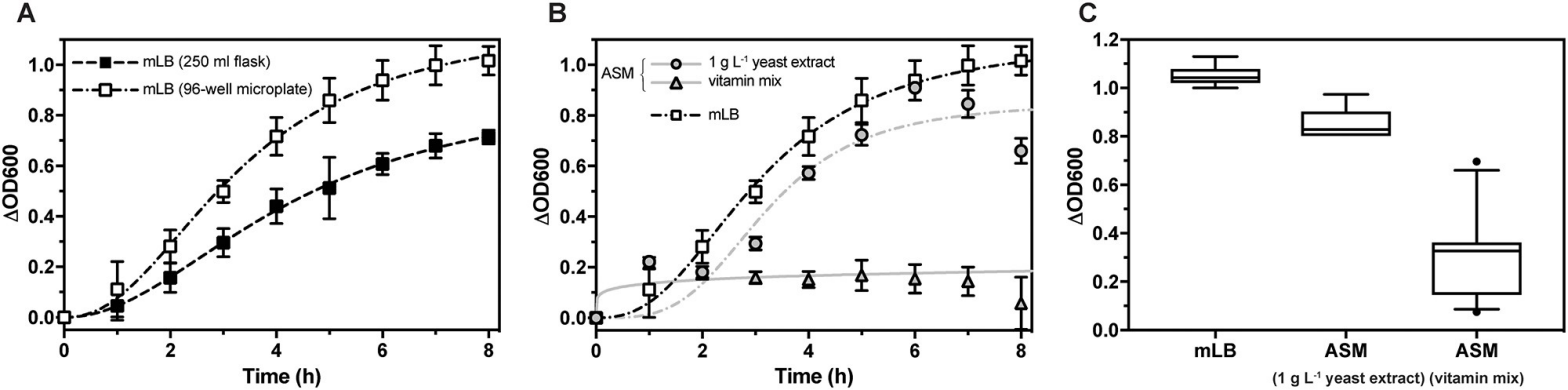
Growth of *G*. *thermoglucosidans* in the complex medium mLB and ASM. (A) *G*. *thermoglucosidans* cultures were grown in mLB in either shake flask format or 96-well microplate format. Points represent the mean change in OD600 of *n* = 12 biological replicates. Error bars represent standard deviation of the mean. The lines represent fits of the data using a one-site specific binding with Hill slope equation. (B) *G*. *thermoglucosidans* cultures were grown in ASM with 10 g L^-1^ glucose supplemented with either 1 g L^-1^ yeast extract or vitamin solution in 96-well microplate format. Points represent the mean change in OD600 of *n* = 6 biological replicates for ASM with 1 g L^-1^ yeast extract or n = 30 biological replicates for ASM with vitamin solution. The lines represent fits of the data using a one-site specific binding with Hill slope equation. (C) Δ_OD600_ after 24 h incubation of *G*. *thermoglucosidans* cultured in 96-well microplates in either mLB, ASM with 1 g L^-1^ yeast extract or ASM with vitamin solution. Boxes represent the 25–75 percentile range, whiskers the 5–95 percentile range and outliers are represented as points.

Growth of *G*. *thermoglucosidans* in the complex media, mLB, was monitored in batch culture in the ubiquitous shake flask format; and to allow for increased experimental throughput, compared to growth in 96-well microplates (Fig 1A). *G*. *thermoglucosidans* cultured in mLB had similar generation times in both shake flask and microplate (flask = 49 min, microplate = 55 min, P = 0.3418) but reached a higher OD600 in microplate. Further experiments were therefore performed in microplate format.

In the defined media, ASM (Zuvasyntha), growth was monitored with 10 g L^-1^ glucose and supplemented with either 1 g L^-1^ yeast extract or a vitamin solution (Fig 1B). The generation time of *G*. *thermoglucosidans* cultured in ASM supplemented with yeast extract was slower than in mLB (100 min). The maximum OD600 occurred at 6 h, due to subsequent culture aggregation and a drop in OD600. The cause of this aggregation, which did not occur in cultures grown in mLB, could be due to the exhaustion of growth substrate which has been demonstrated to cause cell death and lysis [36]. Growth of cultures in ASM supplemented with a vitamin mix was inconsistent. No growth was observed between 0–8 h (Fig 1B), and after 24 h OD600 was highly variable (Fig 1C). These results indicated that a new defined media recipe was required to allow for investigation of carbohydrate utilisation without complex bionutrients such as yeast extract being required for growth.

### A design of experiments approach to defined media formulation

**Fig 2:**
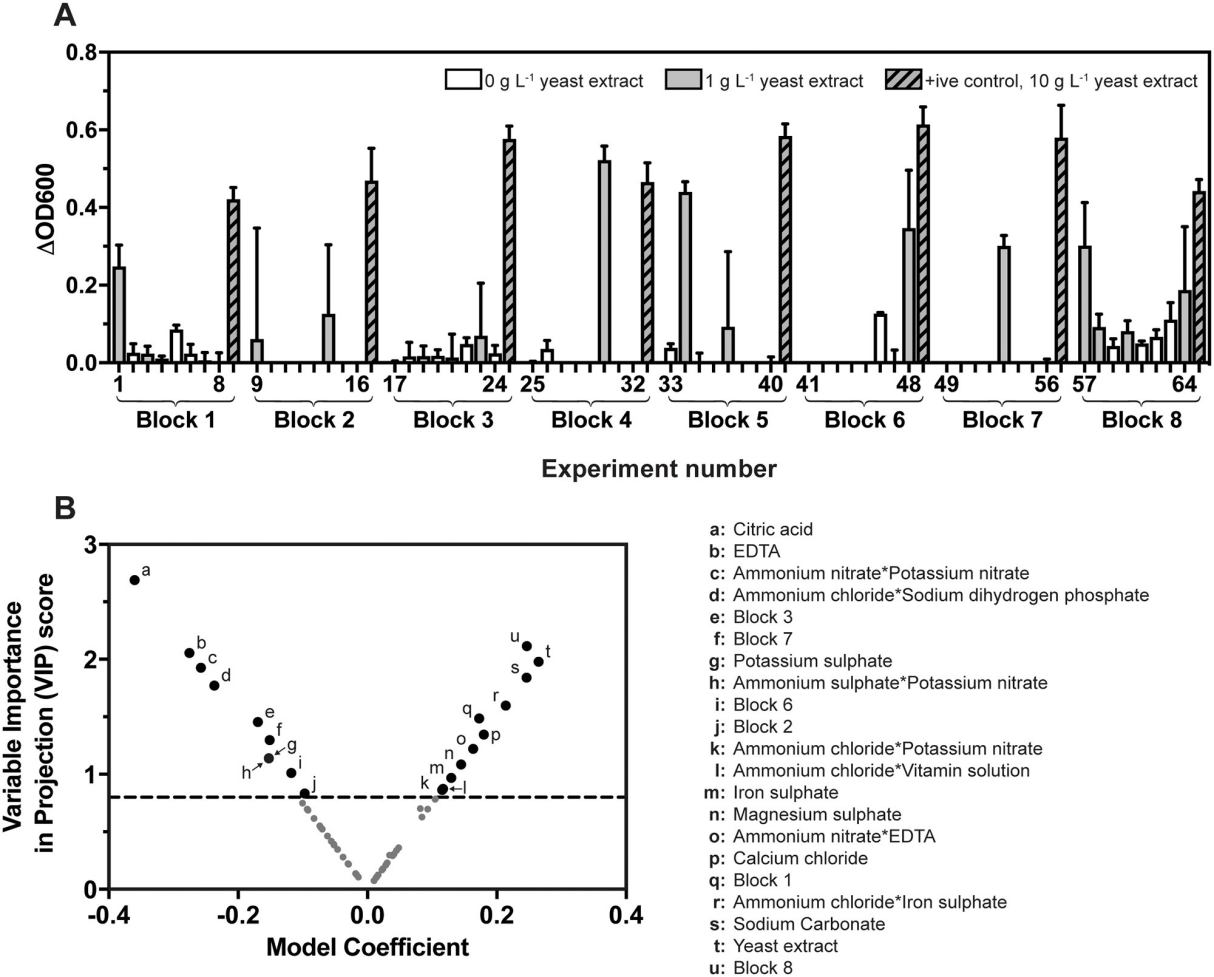
First iteration of Design of Experiments for the development of a defined *Geobacillus* growth medium. (A) Δ_OD600_ after 24 h incubation of *G*. *thermoglucosidans* cultured in each of the 64 media formulations. Bars represent the mean Δ_OD600_ of *n* = 3 starter cultures, with standard deviation error bars shown unless hidden by the bar. Positive controls, *G*. *thermoglucosidans* cultured in 10 g L^-1^ yeast extract, were included on each 96-well microplate to account for batch effects. (B) The relationship between each of the defined media ingredients and culture Δ_OD600_ was analysed using a Partial Least Squares (PLS) model. The Variable Importance in Projection (VIP) score for each of the defined experimental factors is plotted against the centred and scaled model coefficient. Factors with a VIP of 0.8 or greater (dashed line) are considered to be important in determining model output. Conversely, factors with a VIP score that is less than 0.8 are candidates for removal from future models [34]. Interactions between factors are indicated by an asterisk (*).

A review of defined media recipes led to the identification of 21 possible media ingredients (S2 Table), and a Design of Experiments approach was used to define 64 media formulations from these ingredients. The 64 treatments were randomly distributed into 8 blocks for testing (S4 Table), and in all instances, media were supplemented with 10 g L^-1^ glucose. 31 out of the 64 defined media formulations resulted in growth of *G*. *thermoglucosidans* cultures after 24 h (Fig 2A); Of these 31 media compositions, 54% contained 1 g L^-1^ yeast extract. Furthermore, 75% of the 16 treatments that fell in the upper quartile of the Δ_OD600_ distribution (i.e. those treatments with the highest growth after 24 h) contained yeast extract. This result is indicative as to why *Geobacillus* has previously been grown in semi-defined media.

Statistical analysis of the data was performed using Partial Least Squares (PLS) regression modelling. Data were modelled using the non-iterative PLS algorithm (NIPALS) and KFold cross validation, with K = 7. Culture Δ_OD600_ after 24 h incubation was used as the response variable, and media ingredients and the defined first order interactions were used as predictor variables (*X*). The optimum model obtained described the relationship between culture Δ_OD600_ and the predictor variables using 4 LVs. The model was capable of explaining 98.94% of the cumulative variation in *Y* and 7.27% of the cumulative variation in *X* and had a Root Mean PRESS of 0.86.

The PLS model indicated that several media ingredients had a significant effect on *G*. *thermoglucosidans* growth (Fig 2B, S6 Table). Perhaps unsurprisingly, yeast extract was predicted to be the media ingredient with the largest positive contribution to *Geobacillus* growth (VIP = 1.9781, coefficient = 0.2646). Conversely, despite its inclusion in previous defined *Geobacillus* growth media [5,37], citric acid was shown to inhibit growth (VIP = 2.896, coefficient = -0.3598). Similarly, EDTA, which was included in the experimental design due to its use in a *G*. *stearothermophilus* defined media [13] and the ubiquitous *E*. *coli* M9 media [27], was also shown to have a significantly inhibitory effect on *G*. *thermoglucosidans* growth (VIP = 2.0562, coefficient = -0.2751). The inhibitory effects of both citric acid and EDTA were hypothesised to be a result of the chelating properties of both chemicals, which may have minimised the availability of essential metal ions in the growth media. Given these inhibitory effects, neither citric acid nor EDTA were included in the second DoE iteration.

Other media ingredients were also identified by the PLS model as being detrimental to *G*. *thermoglucosidans* growth. Potassium sulphate, for example, was shown to inhibit growth (Fig 2B, S6 Table) and was therefore removed from the second DoE iteration. Additionally, although the VIP score returned by ammonium nitrate did not exceed the 0.8 threshold score, six of the nine predictors that included ammonium nitrate returned negative model coefficients. As such, ammonium nitrate was also removed from the second iteration of DoE.

Counterintuitively, treatments that included either of the two phosphate salts returned small, but negative PLS coefficients, both as individual factors and as first order interactions with other factors (coefficients: -0.0463, -0.0737, -0.0170, -0.2369 and -0.00515), suggesting they were inhibiting *G*. *thermoglucosidans* growth. As the monobasic phosphate and dibasic phosphate were added as individual salts, the random combinations of factors generated by the DoE software could have resulted in treatments where the pH of the resulting media was raised or lowered, respectively. Given the importance of phosphate in cellular processes such as the production of nucleic acids, phospholipid bilayers and ATP, a complete removal of phosphate salts from the growth media was judged likely to be detrimental. Therefore, the two salts were combined into a single 1 M, pH 7 phosphate buffer. The inclusion of MOPS buffer was also found to be slightly inhibitory (VIP = 0.4635, coefficient = -0.062), so was subsequently replaced with a pH 7 Bis-Tris buffer which has previously been used in a *G*. *thermoglucosidans* media [5].

Finally, the PLS model also suggested that batch effects were contributing significantly to culture growth; blocks 1, 2, 3, 6, 7 and 8 all returned VIP scores of greater than 0.8. This could be due to a clustering of successful experiments within blocks despite the random distribution assigned by the DoE software, for example in block 8 where all eight experiments led to growth (Fig 2A). However, an ordinary one-way ANOVA showed that growth of positive controls was significantly different between the blocks at the 5% significance level (F = 6.317, P = 0.0011). Due to equipment failure, three blocks had to be repeated and therefore required fresh batches of 10 g L^-1^ yeast extract for growth of the positive controls. This technical source of error could be contributing differentially to growth of the positive controls between experimental groups.

In total, nine media ingredients from the first iteration were retained in a second iteration of media development. The amino acid and vitamin solutions, as well as the four mLB trace elements were added to all experimental treatments at their 1x concentrations, as these media ingredients were identified as positively contributing to *G*. *thermoglucosidans* growth (Fig 2B, S6 Table). The trace metal solution, which had a slightly inhibitory effect on growth (VIP = 0.5407, coefficient = -0.0723) was also retained, as the cations in the comprised the solution were not accounted for in any of the other media ingredients.

**Fig 3:**
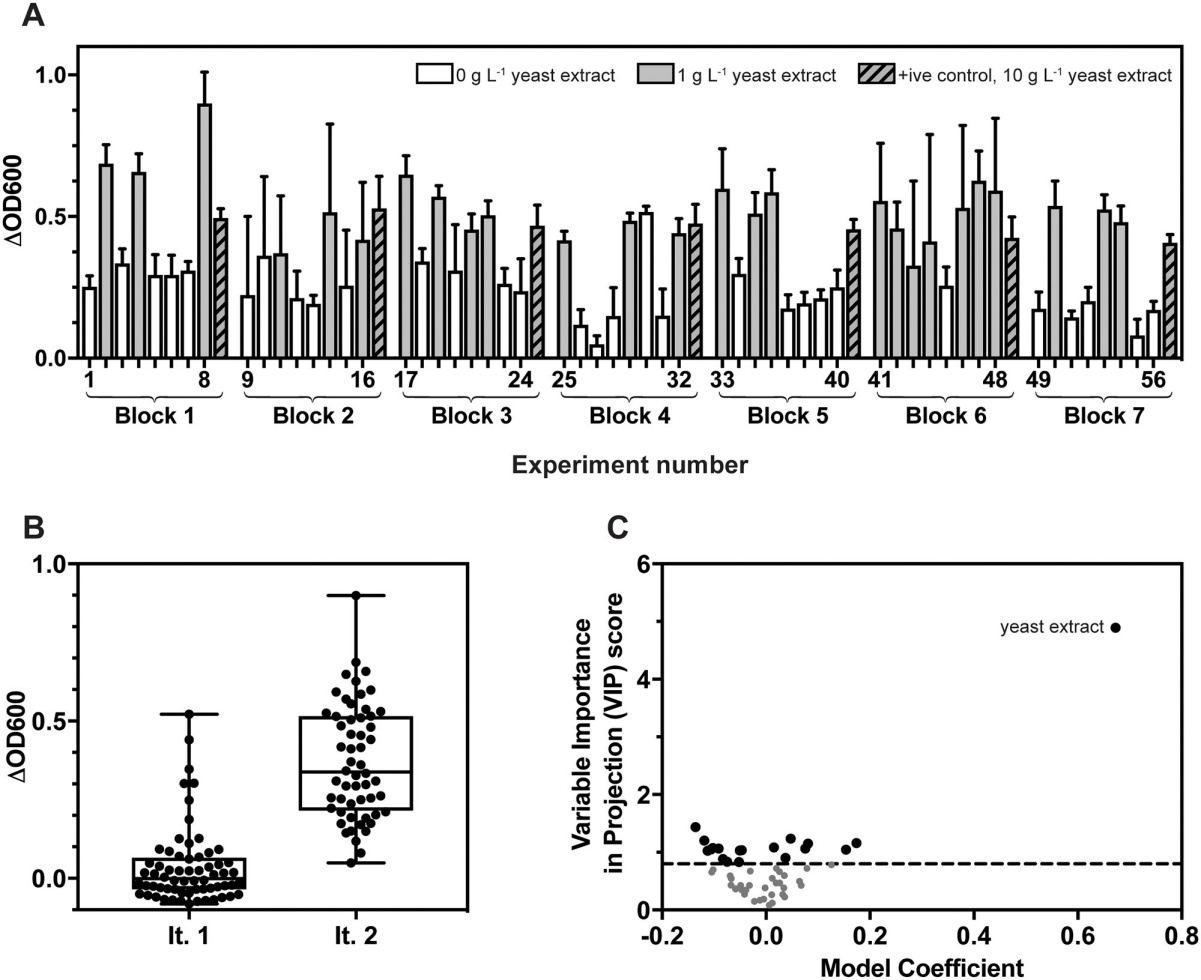
Second iteration of defined media development. (A) Δ_OD600_ after 24 h incubation of *G*. *thermoglucosidans* cultured in each of the 56 defined media formulations. Bars represent the mean Δ_OD600_ of *n* = 3 starter cultures, with standard deviation error bars shown unless hidden by the bar. Positive controls, *G*. *thermoglucosidans* cultured in 10 g L^-1^ yeast extract, were included on each 96-well microplate to account for batch effects. (B) Box and whisker plots showing the distributions of *G*. *thermoglucosidans* growth observed in the first and second iterations of defined media development. Points represent the mean Δ_OD600_ of *G*. *thermoglucosidans* cultures grown in each of the 120 defined media recipes. (C) Modelling of the relationship between the defined media compounds and culture Δ_OD600_ using PLS. The dashed line represents the cut-off VIP score of 0.8.

The custom design platform of JMP software was used to define a 56-treatment experimental design (S5 Table) that provided estimates for all main effects for the nine identified media ingredients and all possible first order interactions. All 56 of these experimental treatments resulted in *G*. *thermoglucosidans* growth after 24 h (Fig 3A), and all but one of the 56 experimental treatments in the second design iteration resulted in culture growth that was above the 75^th^ percentile of Δ_OD600_ seen in the first iteration (Fig 3B), demonstrating an improvement in media composition between the iterations. There was no significant difference in the growth of the positive controls between blocks, as measured by ordinary one-way ANOVA at the 5% significance level (F = 2.261, P = 0.0599), suggesting that the technical sources of error seen during iteration 1 had been successfully reduced.

Statistical analysis of the data was performed using Partial Least Squares (PLS) regression modelling. The optimum model obtained described the relationship between culture Δ_OD600_ and the predictor variables (*i*.*e*. the nine identified media ingredients and all possible first order interactions) using 2 LVs, and was capable of explaining 93.11% of the cumulative variation in *Y* and 6.92% of the cumulative variation in *X*. The Root Mean PRESS of the optimum obtained model was 0.8962.

As in the first iteration, yeast extract was shown to be the media ingredient with the strongest positive impact on *G*. *thermoglucosidans* growth (Fig 3C). Three media ingredients returned negative model coefficients (ammonium sulphate, sodium chloride and sodium carbonate) however their VIP scores were lower than the cut-off score of 0.8, indicating that these were non-significant (S7 Table). The remaining model coefficients for individual factors were positive, indicating that no individual media ingredient significantly inhibited *G*. *thermoglucosidans* growth. In addition, a number of first order interactions between ingredients were predicted to be have a small, but inhibitory effect.

**Fig 4:**
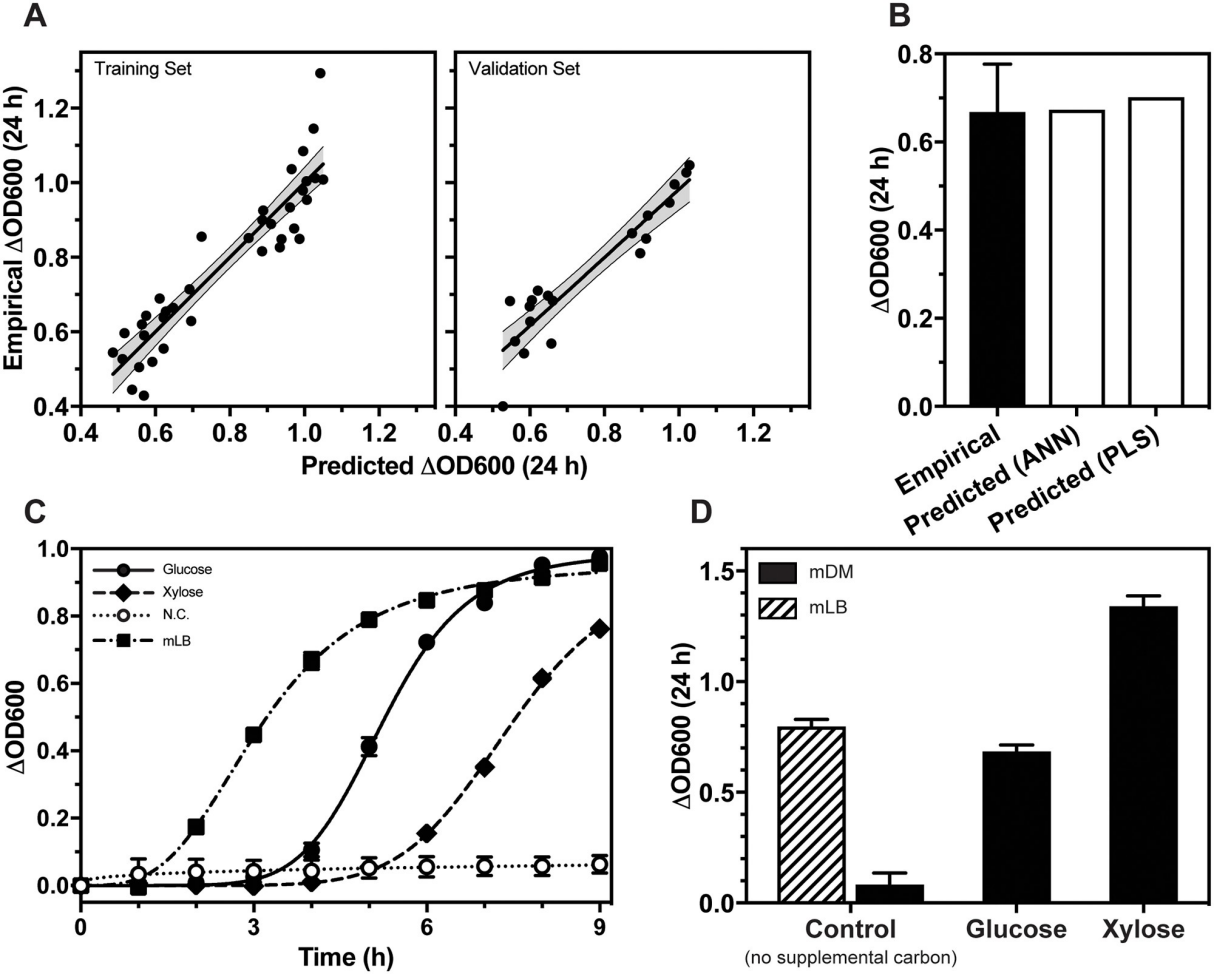
Using an artificial neural network (ANN) model to develop a mathematically defined medium (mDM) for *Geobacillus* growth. (A) Graphs representing empirically determined Δ_OD600_ generated during the second iteration versus predicted Δ_OD600_ generated from a weighted ensemble of the prediction formulae of the 15 regression models. The ANN returned R^2^ values of 0.854 when applied to a training data set and 0.866 when applied to a validation set. (B) A graph showing the expected Δ_OD600_ after 24 hours for the best performing media formulation predicted by the ANN models and the PLS model, and the empirically measured Δ_OD600_ for the ANN media formulation. The empirical bar represents the mean Δ_OD600_ of *n* = 3 with the error bar representing the standard deviation of the mean. (C) *G*. *thermoglucosidans* cultures incubated in 96-well microplates at 60 °C, with shaking at 800 rpm. Cultures were grown in mDM supplemented with either: no additional carbon, 10 g L^-1^ glucose or 10 g L^-1^ xylose. Points represent the mean Δ_OD600_ of *n* = 3 starter cultures, with standard deviation error bars shown unless hidden by the point. Lines represent fits of the data using a one-site specific binding with Hill slope equation. (D) Δ_OD600_ after 24 h incubation. Bars represent the mean Δ_OD600_ of *n* = 3 starter cultures, with standard deviation error bars shown unless hidden by the bar.

The data were also modelled using Stepwise regression. All possible linear regression models were fit, allowing a maximum of six terms per model with Heredity Restriction. The goodness-of-fit of each of the resulting 9,531,039 models was assessed using the Akaike Information Criterion (AICc). For each candidate model (*i*), the Kullback-Leibler distance from the optimum model (*i*.*e*. the model with the smallest AICc) was calculated as Δ_*i*_ = *AICc*_*i*_*—AICc*_*min*_. Models were selected for further interrogation when Δ_*i*_ was less than 2.0 [35]. 15 models were identified as providing strong fits of the data (S8 Table). A weighted ensemble of these 15 models was subsequently generated using an ANN that predicted culture Δ_OD600_ as a function of the prediction formulae of the 15 regression models. The ANN returned R^2^ values of 0.854 when applied to a training data set and 0.866 when applied to a validation set, indicating a good fit of the training data and strong predictive power when applied to novel data (Fig 4A).

To further validate the ANN and to develop a chemically defined minimal media, the simulator function of the JMP software was used to generate 5,000 potential media formulations. In each of the candidate media, the concentrations of the eight chemically defined media ingredients from the second DoE iteration were randomly assigned. In all instances, the amount of yeast extract was set to zero. The ANN and PLS models were subsequently used to make predictions of *G*. *thermoglucosidans* culture Δ_OD600_ after 24 h for each of the 5,000 formulations. The media formulation (Table 1) that was predicted by the ANN to result in the highest *G*. *thermoglucosidans* OD600 after 24 h incubation was tested *in vivo*. The empirically measured Δ_OD600_ was shown to correlate strongly with the values predicted by both the ANN and PLS models (Fig 4B).

**Table 1:** Final mathematically defined media recipe.

Chemical	Final concentration
Ammonium chloride	18.5 mM
Ammonium sulphate	23.7 mM
Potassium nitrate	19 mM
Sodium carbonate	0.63 mM
Sodium chloride	3.03 mM
Dipotassium hydrogen phosphate	33.6 mM
Sodium di-hydrogen phosphate	21.5 mM
Bis-Tris-HCl pH7	43.3 mM
Calcium chloride	0.91 mM
Iron sulphate	0.04 mM
Magnesium sulphate	0.59 mM
NTA sodium salt	1.05 mM
Biotin	19 μM
MEM vitamin solution (Thermo Scientific)	1 x
MEM amino acids (Thermo Scientific)	1 x
MEM non-essential amino acids (Thermo Scientific)	1 x
Trace metal solution (S3 Table)	1 x
Glucose	10 g L^-1^

Concentrations of the components for the best performing media formulation predicted by the ANN model from the second iteration. Note: the combination of the two phosphate salts give a final pH of 7.

Growth in the newly defined mDM was compared to growth in the complex media mLB (Fig 4C). A longer lag phase was observed for cultures in mDM, although after 8 h, growth was comparable to that in mLB, and in log phase doubling time in mDM was shorter (38 min, SD = 3.6). To determine if growth of *G*. *thermoglucosidans* could be supported by an alternative carbon source, the glucose used during the development of mDM was replaced with 10 g L^-1^ xylose (Fig 4C). Cultures grown with xylose exhibited a longer lag phase compared to cultures grown with glucose, a slightly longer doubling time (47 min, SD = 11.6) but after 24 h growth Δ_OD600_ was significantly higher (Fig 4D).

### Analysis of carbon utilisation of *Geobacillus thermoglucosidans*

To assess the capability of the newly developed mDM to facilitate the profiling of carbon utilisation, growth of *G*. *thermoglucosidans* was investigated in the presence of a variety of soluble lignocellulose-based carbohydrates, at a final concentration of 10 g L^-1^. Although microplate culture was invaluable in facilitating the throughput required for a DoE approach to media development, culture growth in microplates may not necessarily be fully representative of growth at larger scales. *G*. *thermoglucosidans* carbohydrate utilisation was therefore assessed in 50 ml batch cultures.

**Fig 5:**
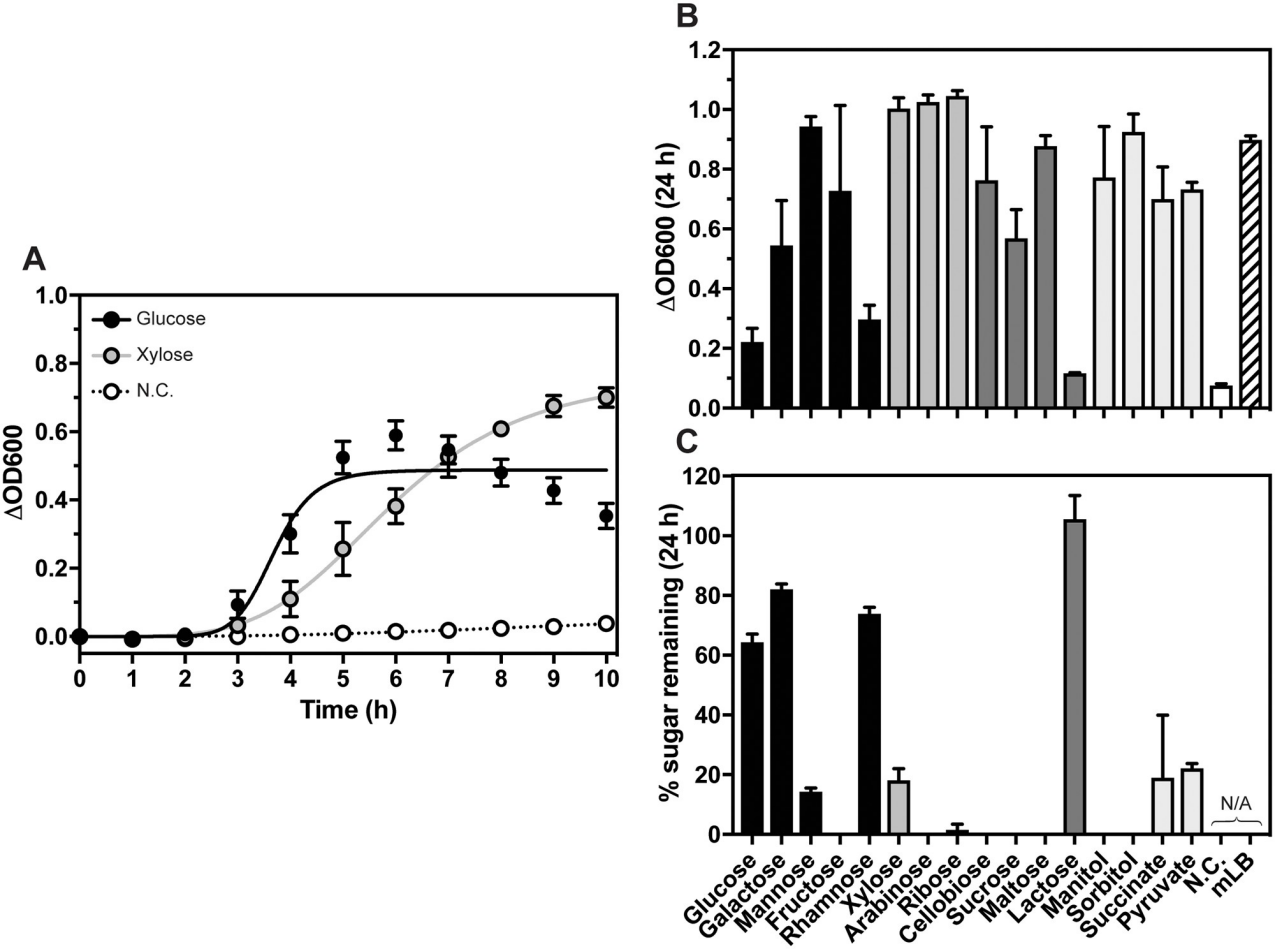
Growth of *G*. *thermoglucosidans* in mDM supplemented with different carbon sources. *G*. *thermoglucosidans* cultures were grown for in 250 ml shake flask format. (A) Growth of *G*. *thermoglucosidans* in mDM supplemented with 10 g L^-1^ Glucose (black) or Xylose (grey), or with no supplemental carbon (N.C., white). Points represent the mean Δ_OD600_ of *n* = 3 starter cultures, with standard deviation error bars shown unless hidden by the point. Lines represent fits of the data using a one-site specific binding with Hill slope equation. (B) Δ_OD600_ after 24 h incubation of *G*. *thermoglucosidans* cultures grown in mDM supplemented with various carbon sources and (C) their associated solvent profiles, as characterised by HPLC, showing the proportion of carbohydrate remaining in the growth media.

*G*. *thermoglucosidans* was capable of utilising five hexose monosaccharides for growth, with final Δ_OD600_ (Fig 5B) and doubling times in log phase (Table 2) dependent on the monosaccharide. The most rapid growth was observed with glucose, the most prevalent carbohydrate in lignocellulose, with a 2 h lag time and a doubling time of 40 min (S1 Fig). However, culture aggregation was observed after 6 h and growth was arrested, resulting in only 36% of the available glucose being utilised (Fig 5C). The pH of the growth media reached pH 6.2 after 24 h and analysis of this media by HPLC demonstrated that the drop in pH was likely due to the production of both lactate and acetate in these cultures (Table 2). This acidification could be the cause of the aggregation observed. Aggregation was also observed in cultures grown with fructose, where both lactate and acetate were excreted into the growth medium.

**Table 2:** Growth characteristics of *G*. *thermoglucosidans* in mDM supplemented with different carbon sources.

Carbohydrate	Doubling time min (SD)	Lactate produced g L^-1^ (SD)	Acetate produced g L^-1^ (SD)
Glucose	40 (2.6)	0.47 (0.09)	0.51 (0.38)
Galactose	167 (2.0)		
Mannose	65 (3.9)		0.16 (0.16)
Fructose	54 (1.7)	0.76 (0.90)	0.31 (0.25)
Rhamnose	N/A	0.33 (0.12)	
Xylose	58 (2.1)		0.17 (0.17)
Arabinose	52 (0.8)		0.43 (0.35)
Ribose	62 (1.5)		0.71 (0.55)
Cellobiose	47 (3.1)		
Sucrose	44 (2.1)	1.18 (0.29)	0.74 (0.560)
Maltose	44 (1.4)		0.27 (0.24)
Lactose	N/A	N/A	N/A
Mannitol	45 (1.2)	1.25 (0.64)	0.35 (0.29)
Sorbitol	67 (1.0)	0.09 (0.15)	0.494 (0.34)
Succinate	80 (12.9)		
Pyruvate	58 (1.8)		

*G*. *thermoglucosidans* cultures were grown for in 250 ml shake flask format. All cultures were grown in mDM supplemented with 10 g L^-1^ of the relevant carbon source. Values represent the mean of *n* = 3 independent starter cultures, with the standard deviation shown in brackets.

Pentose monosaccharides, including xylose, the major constituent of hemicellulose, were also capable of sustaining *G*. *thermoglucosidans* growth. Although cultures grown in xylose, arabinose and ribose had longer doubling times than in glucose (Table 2, S1 Fig), all reached final densities that were significantly greater than even cultures grown in mLB (Fig 5B), as determined by ordinary one-way ANOVA (F = 21.2, P = 0.0004). Growth in all three pentose monosaccharides resulted in acetate production (Table 2) and no aggregation was observed.

In addition to the discussed monosaccharides, *G*. *thermoglucosidans* was capable of utilising certain disaccharides (Fig 5B, Table 2 and S1 Fig). Growth rates in cellobiose, sucrose and maltose were comparable to glucose as determined by ordinary one-way ANOVA (F = 3.934, P = 0.0539). Aggregation was observed in cultures grown with cellobiose and sucrose, although in the presence of the former HPLC analysis did not detect lactate or acetate production (Table 2) and there was no drop in pH. *G*. *thermoglucosidans* was unable to utilise lactose.

Finally, *G*. *thermoglucosidans* cultures were also grown in mDM supplemented with uronic acids, sugar alcohols and carboxylic acids, minor constituents of lignocellulose. Glucuronic and galacturonic acids were unable to sustain culture growth (data not shown) but *G*. *thermoglucosidans* was able to utilise mannitol, sorbitol, succinate and pyruvate (Fig 5B, Table 2 and S1 Fig) and no aggregation was observed.

## Discussion

The growth characteristics of *G*. *thermoglucosidans* were investigated in both complex and defined media in order to inform the development of this bacteria as an industrial host. This study highlighted the need for a new defined media recipe capable of sustaining *G*. *thermoglucosidans* growth the absence of complex bionutrients which would otherwise confound efforts to metabolically profile the species. The DoE approach to developing a defined media allowed a statistically robust investigation into media formulation including the use of the relatively new technology of artificial neural networks (ANNs). In this study, ANNs were not used to directly predict an optimised media formulation. Instead, an ANN was used to create a weighted ensemble of 15 linear regression models, each of which predicted Δ_OD600_ as a function of various media components (S8 Table). Ensembling is a commonly accepted approach, analogous to calculating a mean value of a continuous measurement of interest from biological replicates with less variance to the true or ideal value; by training multiple models on the same task and then combining the outputs of these models, ensembling attempts to exploit information about the design space that might have otherwise been judged redundant if only a single “best” performing model were isolated [38–40]. Theoretically, ANNs could have been trained that predicted Δ_OD600_ directly as a function of the identified media components [41–43]. However, the “black-box” nature of ANNs complicates their use in assessing the contribution of individual media components to culture growth. The combination of linear regression techniques, through which the effect of individual media components can be calculated, and an ANN to create a non-linear ensemble, was shown in this investigation to result in a model which could be applied to both accurately predict culture growth in an optimised growth medium and also improve our fundamental understanding of the contribution of individual media components to culture growth.

Despite reports that indicate *Geobacilli* are able to utilise a variety of carbohydrates for growth [3,6,23], the species are also known to have complex nutritional requirements that is often met by the addition of tryptone or yeast extract [15,44]. The DoE approach has investigated the complex nutritional design space as a whole and has allowed for production of a defined media recipe that fulfils the specific requirements of *G*. *thermoglucosidans* DSM 2542 (NCIMB 1195). The DoE approach has also highlighted a surprising result, the inhibitory effect of citric acid. The addition of citrate in many previous semi-defined media, including ASM, may explain why these recipes have all required either yeast extract or tryptone for growth.

Using this defined media, *G*. *thermoglucosidans* has been shown to utilise both hexose monosaccharides and disaccharides for growth with production of lactate, acetate or both, dependent on the carbohydrate. As stated above the aggregation observed in the presence of some carbohydrates, was not due to exhaustion of substrate, but was accompanied by a drop in pH and specifically, the production of lactate. It has been previously observed that during low redox conditions an upregulation of glycolysis is accompanied by a down regulation of the TCA cycle and a switch to fermentative metabolism [45]. In addition in other thermophilic bacteria, lactate production results from the activation of L-lactate dehydrogenase due to an increase in intracellular concentrations of fructose-1,6-diphosphate, a key intermediate in glycolysis [13]. However, it is unclear why production of lactate and the resulting drop in pH leads to *G*. *thermoglucosidans* culture aggregation.

## Conclusion

The mathematically defined medium developed in this work using a design of experiments approach should allow future metabolic profiling studies to be performed with *G*. *thermoglucosidans* (DSM2542) thereby allowing a more comprehensive understanding of metabolism and providing a better starting point for metabolic engineering of this industrially important microorganism.

## Supporting information

S1 Table Bionutrient requirements of *Geobacilli*. A review of the literature summarising semi-defined media used to grow various species of *Geobacillus*, highlighting the concentration of complex bionutrients added to these media.
(PDF)

S2 Table Defined media ingredients. Commonly used ingredients found in defined media recipes for a variety of microorganisms identified in the literature with maximum concentrations used during the DoE experiments.
(PDF)

S3 Table 100x trace metal solution. Composition of the trace metal solution developed based on the reviewed defined media recipes.
(PDF)

S4 Table First iteration of Design of Experiments for the development of a defined *Geobacillus* growth medium. The 64 media formulations generated by the DoE Fractional Factorial design combining random combinations of the 21 factors identified from the literature search of defined media.
(PDF)

S5 Table Second iteration of defined media development. The 56 media formulations generated by the Custom Design platform of the JMP software. The nine media ingredients were identified in the first iteration as influencing *G*. *thermoglucosidans* growth.
(PDF)

S6 Table. PLS Variable Importance in Projection (VIP) scores and centred and scaled model coefficients for media ingredients and interactions (*) from the first DoE iteration. Sorted in descending order of VIP (*i*.*e*. those factors that are predicted to have the strongest influence on final culture densities are listed first).
(PDF)

S7 Table PLS Variable Importance in Projection (VIP) scores and centred and scaled model coefficients for media ingredients and interactions (*) from the second DoE iteration. Sorted in descending order of VIP (*i*.*e*. those factors that are predicted to have the strongest influence on final culture densities are listed first).
(PDF)

S8 Table Measures of fit for 15 stepwise regression models of the results of the second DoE iteration. All possible linear regression models were fit to the data, with Heredity Restriction and a maximum of 6 terms per model. The goodness-of-fit of each of the resulting 9,531,039 models was assessed using the Akaike Information Criterion (AICc). For each candidate model (*i*), the Kullback-Leibler distance from the optimum model (*i*.*e*. the model with the smallest AICc) was calculated as Δ_*i*_ = *AICc*_*i*_*—AICc*_*min*_. Models were selected for further interrogation when Δ_*i*_ was less than 2.0 (Burnham *et al*., 2010). The 15 selected models are listed in the table in ascending order of Δ_*i*._
(PDF)

S1 Fig Growth of G. thermoglucosidans in the defined medium mDM supplemented with various carbon sources. *G*. *thermoglucosidans* cultures were grown in 250 ml flask format in mDM supplemented with 10 g L^-1^ of either (A) hexose monosaccharides; (B) pentose monosaccharides; (C) disaccharides and (D) sugar alcohols, alpha-keto acid and dicarboxylic acid. All graphs show growth curves for *G*. *thermoglucosidans* cultures grown in mLB and mDM with no additional carbon source for comparison. In all cases, cultures were inoculated to an initial OD600 of 0.04 using starter cultures prepared by resuspending *G*. *thermoglucosidans* biomass taken from a confluent agar plate in 5 g L^-1^ yeast extract solution. Cultures were incubated at 60 °C, with shaking at 220 rpm. Points represent the mean change in OD600 of *n* = 3 biological replicates. Standard deviation error bars are shown, unless hidden by the points. The lines represent fits of the data using a one-site specific binding with Hill slope equation.
(PDF)
