# Supplementary material for: A design of experiments approach for the rapid formulation of a chemically defined medium for metabolic profiling of industrially important microbes
Source: PLoS One. 2019 Jun 12;14(6):e0218208. doi: 10.1371/journal.pone.0218208 (PMC6561596; doi:10.1371/journal.pone.0218208)
Supplement: S1 Table — A review of the literature summarising semi-defined media used to grow various species of Geobacillus, highlighting the concentration of complex bionutrients added to these media. (PDF) [file pone.0218208.s001.pdf]

Species	Media	Carbon source	Bionutrient	Reference
G. thermoglucosidasius	TB-ASM	0.1 g L^{-1} cellobiose, glucose or xylose	10 g L^{-1} tryptone, 1 g L^{-1} yeast extract	[7]
Geobacillus strain LC300	Growth medium	2 g L^{-1} xylose	0.05 g L^{-1} yeast extract	[9]
G. thermoglucosidasius	ASYE	20 g L^{-1} glucose	5 g L^{-1} yeast extract	[5]
G. thermoglucosidasius	USYE	30 g L^{-1} glucose	10 g L^{-1} yeast extract	[5]
G. thermoglucosidasius	USYE	10 g L^{-1} glucose	1 g L^{-1} tryptone, 1 g L^{-1} yeast extract	[5]
G. thermoglucosidasius	USYE	30 g L^{-1} cellobiose or xylose	10 g L^{-1} yeast extract	[5]
G. thermoglucosidasius	TMM	2 g L^{-1} glucose, xylose, arabinose or cellobiose	0.2 or 0.5 g L^{-1} yeast extract	[10]
G. thermoglucosidasius	CBM	N/A	4 g L^{-1} casein hydrolysate	[8]
G. thermoglucosidasius	ASYE	20 g L^{-1} glucose	10 g L^{-1} yeast extract	[8]
G. kaustophilus	MM	Various	1 g L^{-1} casamino Acids	[11]
G. kaustophilus	MY	10 g L^{-1} maltose	10 g L^{-1} yeast extract	[11]
G. thermoglucosidasius	TMLM	10 g L^{-1} glucose	1 g L^{-1} yeast extract	[37]
G. thermoglucosidasius	USYE	30 g L^{-1} glucose or cellobiose	1 g L^{-1} yeast extract	[37]

Table S1. Bionutrient requirements of *Geobacilli.*
A review of the literature summarising semi-defined media used to grow various species of *Geobacillus*, highlighting the concentration of complex bionutrients added to these media.
