# Supplementary material for: A design of experiments approach for the rapid formulation of a chemically defined medium for metabolic profiling of industrially important microbes
Source: PLoS One. 2019 Jun 12;14(6):e0218208. doi: 10.1371/journal.pone.0218208 (PMC6561596; doi:10.1371/journal.pone.0218208)
Supplement: S2 Table — Commonly used ingredients found in defined media recipes for a variety of microorganisms identified in the literature with maximum concentrations used during the DoE experiments. (PDF) [file pone.0218208.s002.pdf]

Chemical	Maximum concentration mM	Reference
Ammonium chloride	50	[13]
Ammonium nitrate	50	This paper
Ammonium sulphate	50	[7]
Potassium sulphate	50	[7]
Potassium nitrate	50	This paper
Sodium carbonate	1	This paper
Sodium chloride	8.6	[27]
Dipotassium hydrogen phosphate	33	This paper
Sodium di-hydrogen phosphate	17	[7]
Urea	50	[5]
Citric acid	10	[5]
MOPS	50	[26]
Calcium chloride	1	[23]
Iron sulphate	0.1	[23]
Magnesium sulphate	1	[23]
NTA sodium salt	1	[23]
EDTA sodium salt	1	[13]

Table S2. Defined media ingredients. Commonly used ingredients found in defined media recipes for a variety of microorganisms identified in the literature with maximum concentrations used during the DoE experiments.
