# Supplementary material for: A design of experiments approach for the rapid formulation of a chemically defined medium for metabolic profiling of industrially important microbes
Source: PLoS One. 2019 Jun 12;14(6):e0218208. doi: 10.1371/journal.pone.0218208 (PMC6561596; doi:10.1371/journal.pone.0218208)
Supplement: S3 Table — Composition of the trace metal solution developed based on the reviewed defined media recipes. (PDF) [file pone.0218208.s003.pdf]

Chemical	Formula	500 x concentration mM	1 x concentration μM
Zinc sulphate	$ZnSO_4.7H_2O$	12.5	25.0
Nickel sulphate	$NiSO_4.6H_2O$	8.52	17.0
Manganese sulphate	$MnSO_4.H_2O$	7.51	15.0
Cobalt sulphate	$CoSO_4.7H_2O$	5.98	9.96
Copper sulphate	$CuSO_4.5H_2O$	2.52	5.05
Disodium molybdate	$Na_2MoO_4.2H_2O$	0.87	1.74
Boric acid	H_3BO_3	0.16	0.32
Sulfuric acid	H_2SO_4	60 (5 ml of 12 M)	120

Table S3. 100x trace metal solution.
Composition of the trace metal solution developed based on the reviewed defined media recipes.
