# Supplementary material for: A design of experiments approach for the rapid formulation of a chemically defined medium for metabolic profiling of industrially important microbes
Source: PLoS One. 2019 Jun 12;14(6):e0218208. doi: 10.1371/journal.pone.0218208 (PMC6561596; doi:10.1371/journal.pone.0218208)
Supplement: S4 Table — The 64 media formulations generated by the DoE Fractional Factorial design combining random combinations of the 21 factors identified from the literature search of defined media. (PDF) [file pone.0218208.s004.pdf]

Experiment	*Block*	*Ammonium chloride*	*Ammonium nitrate*	*Ammonium sulphate*	*Potassium sulphate*	*Potassium nitrate*	*Sodium carbonate*	*Sodium chloride*	*Dipotassium hydrogen phosphate*	*Sodium dihydrogen phosphate*	*Urea*	*Citric acid*	*MOPS*	*Calcium chloride*	*Iron sulphate*	*Magnesium sulphate*	*NTA*	*EDTA*	*Trace metals 500x*	*Vitamins 100x*	*Amino acids 100x*	*Yeast extract*
1	1	0	50	50	50	0	1	0	0	17	0	0	50	1	0	1	0	0	0	1	0	0.1
2	1	50	50	50	50	0	0	8.6	33	0	0	10	0	0	0.1	1	0	1	1	1	0	0
3	1	50	0	50	0	50	0	8.6	0	17	0	10	0	1	0	0	1	0	1	0	0	0.1
4	1	0	0	50	0	50	1	0	33	0	0	0	50	0	0.1	0	1	1	0	0	0	0
5	1	0	50	0	0	50	0	8.6	33	0	50	0	50	1	0	1	0	0	1	0	1	0
6	1	50	0	0	50	0	1	0	33	0	50	10	0	1	0	0	1	0	0	1	1	0
7	1	50	50	0	0	50	1	0	0	17	50	10	0	0	0.1	1	0	1	0	0	1	0.1
8	1	0	0	0	50	0	0	8.6	0	17	50	0	50	0	0.1	0	1	1	1	1	1	0.1
9	2	0	0	50	50	50	1	8.6	33	17	50	0	0	0	0	0	0	0	1	1	1	0.1
10	2	50	50	50	0	0	0	0	33	17	50	10	50	0	0	1	1	0	0	0	1	0.1
11	2	50	0	0	0	0	1	8.6	33	17	0	10	50	1	0.1	0	0	1	1	0	0	0.1
12	2	50	50	0	50	50	1	8.6	0	0	0	10	50	0	0	1	1	0	1	1	0	0
13	2	0	50	50	0	0	1	8.6	0	0	50	0	0	1	0.1	1	1	1	1	0	1	0
14	2	0	50	0	50	50	0	0	33	17	0	0	0	1	0.1	1	1	1	0	1	0	0.1
15	2	0	0	0	0	0	0	0	0	0	0	0	0	0	0	0	0	0	0	0	0	0
16	2	50	0	50	50	50	0	0	0	0	50	10	50	1	0.1	0	0	1	0	1	1	0
17	3	50	0	50	50	0	0	8.6	33	0	0	0	50	1	0	1	0	1	0	0	1	0.1
18	3	50	50	50	0	50	0	8.6	0	17	0	0	50	0	0.1	0	1	0	0	1	1	0
19	3	0	50	50	0	50	1	0	33	0	0	10	0	1	0	0	1	1	1	1	1	0.1
20	3	0	0	50	50	0	1	0	0	17	0	10	0	0	0.1	1	0	0	1	0	1	0
21	3	50	50	0	50	0	1	0	33	0	50	0	50	0	0.1	0	1	0	1	0	0	0.1
22	3	50	0	0	0	50	1	0	0	17	50	0	50	1	0	1	0	1	1	1	0	0
23	3	0	0	0	0	50	0	8.6	33	0	50	10	0	0	0.1	1	0	0	0	1	0	0.1
24	3	0	50	0	50	0	0	8.6	0	17	50	10	0	1	0	0	1	1	0	0	0	0
25	4	0	50	0	0	0	0	0	0	0	0	10	50	1	0.1	0	0	0	1	1	1	0.1
26	4	50	0	50	0	0	0	0	33	17	50	0	0	1	0.1	1	1	0	1	1	0	0
27	4	0	0	50	0	0	1	8.6	0	0	50	10	50	0	0	1	1	1	0	1	0	0.1
28	4	0	0	0	50	50	0	0	33	17	0	10	50	0	0	1	1	1	1	0	1	0
29	4	50	50	0	0	0	1	8.6	33	17	0	0	0	0	0	0	0	1	0	1	1	0
30	4	50	0	0	50	50	1	8.6	0	0	0	0	0	1	0.1	1	1	0	0	0	1	0.1
31	4	0	50	50	50	50	1	8.6	33	17	50	10	50	1	0.1	0	0	0	0	0	0	0
32	4	50	50	50	50	50	0	0	0	0	50	0	0	0	0	0	0	1	1	0	0	0.1
33	5	50	0	0	50	0	0	8.6	0	17	50	0	50	0	0.1	1	0	0	0	0	0	0
34	5	50	0	50	0	50	1	0	33	0	0	0	50	0	0.1	1	0	0	1	1	1	0.1
35	5	0	50	50	50	0	0	8.6	33	0	0	10	0	0	0.1	0	1	0	0	0	1	0.1
36	5	0	0	0	50	0	1	0	33	0	50	10	0	1	0	1	0	1	1	0	0	0.1
37	5	50	50	0	0	50	0	8.6	33	0	50	0	50	1	0	0	1	1	0	1	0	0.1
38	5	0	50	0	0	50	1	0	0	17	50	10	0	0	0.1	0	1	0	1	1	0	0
39	5	50	50	50	50	0	1	0	0	17	0	0	50	1	0	0	1	1	1	0	1	0
40	5	0	0	50	0	50	0	8.6	0	17	0	10	0	1	0	1	0	1	0	1	1	0
41	6	0	50	50	0	0	0	0	33	17	50	10	50	0	0	0	0	1	1	1	0	0
42	6	50	50	0	50	50	0	0	33	17	0	0	0	1	0.1	0	0	0	1	0	1	0
43	6	50	0	0	0	0	0	0	0	0	0	0	0	0	0	1	1	1	1	1	1	0.1
44	6	0	0	50	50	50	0	0	0	0	50	10	50	1	0.1	1	1	0	1	0	0	0.1
45	6	50	0	50	50	50	1	8.6	33	17	50	0	0	0	0	1	1	1	0	0	0	0
46	6	0	0	0	0	0	1	8.6	33	17	0	10	50	1	0.1	1	1	0	0	1	1	0
47	6	0	50	0	50	50	1	8.6	0	0	0	10	50	0	0	0	0	1	0	0	1	0.1
48	6	50	50	50	0	0	1	8.6	0	0	50	0	0	1	0.1	0	0	0	0	1	0	0.1
49	7	50	50	50	0	50	1	0	33	0	0	10	0	1	0	1	0	0	0	0	0	0
50	7	0	0	50	50	0	0	8.6	33	0	0	0	50	1	0	0	1	0	1	1	0	0
51	7	0	50	50	0	50	0	8.6	0	17	0	0	50	0	0.1	1	0	1	1	0	0	0.1
52	7	0	50	0	50	0	1	0	33	0	50	0	50	0	0.1	1	0	1	0	1	1	0
53	7	0	0	0	0	50	1	0	0	17	50	0	50	1	0	0	1	0	0	0	1	0.1
54	7	50	0	50	50	0	1	0	0	17	0	10	0	0	0.1	0	1	1	0	1	0	0.1
55	7	50	0	0	0	50	0	8.6	33	0	50	10	0	0	0.1	0	1	1	1	0	1	0
56	7	50	50	0	50	0	0	8.6	0	17	50	10	0	1	0	1	0	0	1	1	1	0.1
57	8	0	50	0	0	0	1	8.6	33	17	0	0	0	0	0	1	1	0	1	0	0	0.1
58	8	50	50	50	50	50	1	8.6	33	17	50	10	50	1	0.1	1	1	1	1	1	1	0.1
59	8	50	50	0	0	0	0	0	0	0	0	10	50	1	0.1	1	1	1	0	0	0	0
60	8	50	0	0	50	50	0	0	33	17	0	10	50	0	0	0	0	0	0	1	0	0.1
61	8	50	0	50	0	0	1	8.6	0	0	50	10	50	0	0	0	0	0	1	0	1	0
62	8	0	50	50	50	50	0	0	0	0	50	0	0	0	0	1	1	0	0	1	1	0
63	8	0	0	0	50	50	1	8.6	0	0	0	0	0	1	0.1	0	0	1	1	1	0	0
64	8	0	0	50	0	0	0	0	33	17	50	0	0	1	0.1	0	0	1	0	0	1	0.1

Table S4. First iteration of Design of Experiments for the development of a defined *Geobacillus* growth medium.

The 64 media formulations generated by the DoE Fractional Factorial design combining random combinations of the 21 factors identified from the literature search of defined media.
