# Supplementary material for: A design of experiments approach for the rapid formulation of a chemically defined medium for metabolic profiling of industrially important microbes
Source: PLoS One. 2019 Jun 12;14(6):e0218208. doi: 10.1371/journal.pone.0218208 (PMC6561596; doi:10.1371/journal.pone.0218208)
Supplement: S5 Table — The 56 media formulations generated by the Custom Design platform of the JMP software. The nine media ingredients were identified in the first iteration as influencing G. thermoglucosidans growth. (PDF) [file pone.0218208.s005.pdf]

Experiment	Block	Ammonium chloride	Ammonium sulphate	Potassium nitrate	Sodium carbonate	Sodium chloride	Phosphate buffer	Urea	Bis-Tris	Yeast extract
1	1	0	50	50	0	0	0	0	0	0
2	1	50	50	0	1	0	0	50	50	0.1
3	1	50	0	50	0	0	50	0	0	0
4	1	0	50	50	1	8.6	0	0	50	0.1
5	1	50	50	50	1	0	50	0	50	0
6	1	0	50	50	1	8.6	50	0	0	0
7	1	50	0	50	1	0	0	50	0	0
8	1	0	50	0	0	0	50	0	50	0.1
9	2	50	50	0	1	0	0	0	0	0
10	2	50	0	0	1	0	50	50	50	0
11	2	0	50	50	1	0	0	0	0	0.1
12	2	50	50	50	0	0	50	50	0	0
13	2	50	0	0	1	8.6	0	0	50	0
14	2	50	0	0	1	0	50	0	50	0.1
15	2	0	0	50	0	0	50	50	50	0
16	2	0	50	0	0	8.6	0	50	0	0.1
17	3	0	0	0	1	0	50	50	50	0.1
18	3	0	0	50	1	8.6	50	50	50	0
19	3	50	0	50	0	0	0	0	50	0.1
20	3	0	0	0	0	8.6	50	0	50	0
21	3	0	50	50	1	8.6	50	50	0	0.1
22	3	0	0	50	0	8.6	50	50	0	0.1
23	3	0	0	0	0	8.6	50	50	0	0
24	3	50	50	50	0	8.6	0	0	50	0
25	4	50	50	50	0	0	50	0	0	0.1
26	4	50	50	0	0	0	0	50	50	0
27	4	0	0	0	1	0	0	0	50	0
28	4	0	0	50	0	8.6	0	0	50	0
29	4	50	50	0	0	8.6	0	0	50	0.1
30	4	50	0	50	1	8.6	0	50	50	0.1
31	4	50	0	0	0	8.6	0	0	0	0
32	4	50	50	0	1	8.6	50	0	0	0.1
33	5	50	50	0	0	8.6	50	50	50	0.1
34	5	50	0	50	0	8.6	0	50	50	0
35	5	50	0	0	0	0	50	50	0	0.1
36	5	50	0	50	0	8.6	50	0	50	0.1
37	5	50	50	0	1	8.6	0	50	50	0
38	5	50	50	0	0	8.6	50	0	0	0
39	5	0	50	50	0	8.6	50	50	50	0
40	5	0	0	0	0	0	0	50	0	0
41	6	0	0	0	0	8.6	0	50	50	0.1
42	6	0	0	0	1	8.6	50	0	0	0.1
43	6	0	0	0	0	0	0	0	0	0.1
44	6	50	50	50	0	8.6	0	50	0	0.1
45	6	0	0	0	1	0	50	0	0	0
46	6	50	0	50	1	8.6	0	0	0	0.1
47	6	0	0	50	1	8.6	0	50	0	0.1
48	6	50	0	0	1	8.6	0	50	0	0.1
49	7	50	0	50	1	8.6	50	50	0	0
50	7	0	0	50	1	0	50	0	50	0.1
51	7	0	50	0	1	0	50	50	0	0
52	7	0	50	50	1	0	0	50	50	0
53	7	0	50	50	0	0	0	50	50	0.1
54	7	50	0	50	1	0	50	50	0	0.1
55	7	0	50	0	1	8.6	0	0	0	0
56	7	0	50	0	1	8.6	50	0	50	0

Table S5. Second iteration of defined media development.
The 56 media formulations generated by the Custom Design platform of the JMP software. The nine media ingredients were identified in the first iteration as influencing *G. thermoglucosidans* growth.
