# Supplementary material for: A design of experiments approach for the rapid formulation of a chemically defined medium for metabolic profiling of industrially important microbes
Source: PLoS One. 2019 Jun 12;14(6):e0218208. doi: 10.1371/journal.pone.0218208 (PMC6561596; doi:10.1371/journal.pone.0218208)
Supplement: S6 Table. PLS — Sorted in descending order of VIP (i.e. those factors that are predicted to have the strongest influence on final culture densities are listed first). (PDF) [file pone.0218208.s006.pdf]

Predictor variable	VIP score	Centred & scaled coefficient		Predictor Variable	VIP score	Centred & scaled coefficient
Citric acid	2.6896	-0.3598		Ammonium chloride*Urea	0.5227	-0.0699
Block[8]	2.1146	0.2463		MOPS	0.4635	-0.062
EDTA	2.0562	-0.2751		Ammonium nitrate	0.419	-0.0561
Yeast extract	1.9781	0.2646		Ammonium nitrate*Sodium carbonate	0.3909	-0.0523
Ammonium nitrate*Potassium nitrate	1.9257	-0.2576		Ammonium nitrate*Dipotassium hydrogen phosphate	0.3852	-0.0515
Sodium carbonate	1.8407	0.2462		Potassium nitrate	0.3605	0.0482
Ammonium chloride*Sodium dihydrogen phosphate	1.7708	-0.2369		Dipotassium hydrogen phosphate	0.3462	-0.0463
Ammonium chloride*Iron sulphate	1.5984	0.2138		Ammonium chloride*Sodium chloride	0.3372	0.0451
Block[1]	1.4855	0.173		Ammonium nitrate*Calcium chloride	0.3071	0.0411
Block[3]	1.4559	-0.1696		Block[4]	0.2963	0.0345
Calcium chloride	1.3461	0.1801		Ammonium chloride*Potassium sulphate	0.2913	0.039
Block[7]	1.2982	-0.1512		Ammonium chloride*Amino acids 100x	0.2809	-0.0376
Ammonium nitrate*EDTA	1.2214	0.1634		NTA(0,1)	0.229	0.0306
Ammonium sulphate*Potassium nitrate	1.141	-0.1526		Ammonium sulphate*Yeast extract	0.2238	-0.0299
Potassium sulphate	1.1389	-0.1523		Potassium sulphate*Yeast extract	0.2186	-0.0292
Magnesium sulphate	1.0842	0.145		Ammonium chloride*Magnesium sulphate	0.2112	0.0282
Block[6]	1.0113	-0.1178		Ammonium chloride*Ammonium sulphate	0.1791	0.024
Iron sulphate	0.9676	0.1294		Ammonium sulphate	0.167	0.0223
Ammonium chloride*Vitamins 100x	0.8741	0.1169		Ammonium chloride*Trace metals 500x	0.1379	-0.0184
Ammonium chloride*Potassium nitrate	0.8636	0.1155		Ammonium chloride*Dipotassium hydrogen phosphate	0.1268	-0.017
Block[2]	0.8322	-0.0969		Vitamins 100x	0.1244	0.0166
Ammonium chloride*Yeast extract	0.785	0.105		Ammonium chloride	0.1082	0.0145
Sodium chloride	0.7826	0.1047		Ammonium chloride*Calcium chloride	0.1056	-0.0141
Ammonium nitrate*Iron sulphate	0.7498	-0.1003		Ammonium chloride*Sodium carbonate	0.0983	0.0132
Block[5]	0.7012	0.0817		Urea	0.0757	0.0101
Ammonium chloride*Ammonium nitrate	0.6977	-0.0933				
Amino acids 100x	0.6966	0.0932				
Ammonium chloride*EDTA	0.687	-0.0919				
Ammonium nitrate*Ammonium sulphate	0.6287	0.0841				
Ammonium chloride*NTA	0.6166	-0.0825				
Sodium dihydrogen phosphate	0.5506	-0.0737				
Trace metals 500x	0.5407	-0.0723				

Table S6. PLS Variable Importance in Projection (VIP) scores and centred and scaled model coefficients for media ingredients and interactions (*) from the first DoE iteration.

Sorted in descending order of VIP (*i.e.* those factors that are predicted to have the strongest influence on final culture densities are listed first).
