# Supplementary material for: A design of experiments approach for the rapid formulation of a chemically defined medium for metabolic profiling of industrially important microbes
Source: PLoS One. 2019 Jun 12;14(6):e0218208. doi: 10.1371/journal.pone.0218208 (PMC6561596; doi:10.1371/journal.pone.0218208)
Supplement: S7 Table — Sorted in descending order of VIP (i.e. those factors that are predicted to have the strongest influence on final culture densities are listed first). (PDF) [file pone.0218208.s007.pdf]

Predictor variable	VIP score	Centred & scaled coefficient		Predictor Variable	VIP score	Centred & scaled coefficient
Yeast extract(0,0.1)	4.8926	0.6728		Ammonium chloride*Yeast extract	0.4708	0.0196
Block[4]	1.4371	-0.1359		Block[5]	0.4708	0.0264
Block[6]	1.2377	0.0473		Sodium carbonate*Bis-Tris	0.4301	-0.0675
Block[7]	1.2033	-0.1189		Ammonium sulphate*Yeast extract	0.4283	0.0678
Phosphate buffer	1.1587	0.1739		Ammonium chloride*Sodium chloride	0.4276	-0.0318
Block[1]	1.1513	0.0809		Ammonium sulphate*Phosphate buffer	0.4257	-0.0489
Sodium chloride*Urea	1.0825	0.0153		Potassium nitrate	0.3852	-0.0017
Potassium nitrate*Urea	1.076	-0.1022		Sodium carbonate*Urea	0.3835	0.0342
Phosphate buffer*Bis-Tris	1.0651	0.0755		Ammonium chloride*Urea	0.3696	-0.0591
Sodium chloride*Bis-Tris	1.064	-0.091		Ammonium sulphate*Potassium nitrate	0.3634	-0.0585
Sodium chloride*Phosphate buffer	1.0485	-0.1062		Sodium chloride	0.3523	-0.0469
Bis-Tris*Yeast extract	1.0452	0.1541		Sodium chloride*Yeast extract	0.3454	-0.0388
Potassium nitrate*Bis-Tris	1.0344	-0.047		Urea*Yeast extract	0.2733	-0.0415
Ammonium sulphate*Sodium carbonate	1.034	-0.0502		Ammonium chloride	0.2696	0.0321
Potassium nitrate*Phosphate buffer	1.0279	-0.1126		Sodium carbonate*Yeast extract	0.2623	0.0112
Potassium nitrate*Sodium carbonate	0.9046	0.0376		Block[2]	0.2243	0.036
Urea*Bis-Tris	0.8839	-0.0828		Sodium carbonate	0.188	-0.0045
Phosphate buffer*Urea	0.8364	-0.075		Ammonium chloride*Bis-Tris	0.1668	-0.0118
Potassium nitrate*Yeast extract	0.8295	-0.0522		Ammonium sulphate*Sodium chloride	0.1508	-0.0225
Bis-Tris	0.7818	0.1258		Sodium carbonate*Sodium chloride	0.1211	0.0122
Phosphate buffer*Yeast extract	0.7227	0.02		Ammonium chloride*Phosphate buffer	0.0855	0.006
Potassium nitrate*Sodium chloride	0.7223	0.0788				
Sodium carbonate*Phosphate buffer	0.6959	-0.1019				
Ammonium sulphate	0.6726	-0.0302				
Ammonium chloride*Sodium carbonate	0.6646	0.0257				
Ammonium chloride*Potassium nitrate	0.6557	-0.1042				
Urea	0.5992	0.0355				
Ammonium sulphate*Urea	0.5921	-0.069				
Ammonium sulphate*Bis-Tris	0.5511	0.0122				
Ammonium chloride*Ammonium sulphate	0.5437	-0.0671				
Block[3]	0.4979	0.0642				

Table S7. PLS Variable Importance in Projection (VIP) scores and centred and scaled model coefficients for media ingredients and interactions (*) from the second DoE iteration.

Sorted in descending order of VIP (*i.e.* those factors that are predicted to have the strongest influence on final culture densities are listed first).
