# Supplementary material for: A design of experiments approach for the rapid formulation of a chemically defined medium for metabolic profiling of industrially important microbes
Source: PLoS One. 2019 Jun 12;14(6):e0218208. doi: 10.1371/journal.pone.0218208 (PMC6561596; doi:10.1371/journal.pone.0218208)
Supplement: S8 Table — All possible linear regression models were fit to the data, with Heredity Restriction and a maximum of 6 terms per model. The goodness-of-fit of each of the resulting 9,531,039 models was assessed using the Akaike Information Criterion (AICc). For each candidate model (i), the Kullback-Leibler distance from the optimum model (i.e. the model with the smallest AICc) was calculated as Δi = AICci—AICcmin. Models were selected for further interrogation when Δi was less than 2.0 (Burnham et al., 2010). The 15 selected models are listed in the table in ascending order of Δi. (PDF) [file pone.0218208.s008.pdf]

Terms Included in Model	Number of Terms	RSquare	RMSE	AICc	Δ_i
Ammonium chloride, Phosphate buffer, Yeast extract, Potassium nitrate*Sodium chloride, Phosphate buffer*Bis-Tris, Phosphate buffer*Yeast extract	6	0.8436	0.0844	-106.362	0
Phosphate buffer, Yeast extract, Potassium nitrate*Sodium chloride, Phosphate buffer*Bis-Tris, Phosphate buffer*Yeast extract, Bis-Tris*Yeast extract	6	0.8435	0.0844	-106.3123	0.0497
Phosphate buffer, Yeast extract, Potassium nitrate*Sodium chloride, Potassium nitrate*Yeast extract, Phosphate buffer*Bis-Tris, Phosphate buffer*Yeast extract	6	0.8434	0.0845	-106.2805	0.0815
Phosphate buffer, Yeast extract, Potassium nitrate*Sodium chloride, Sodium chloride*Phosphate buffer, Phosphate buffer*Bis-Tris, Phosphate buffer*Yeast extract	6	0.8411	0.0851	-105.4411	0.9209
Phosphate buffer, Yeast extract, Potassium nitrate*Sodium chloride, Phosphate buffer*Bis-Tris, Phosphate buffer*Yeast extract	5	0.8331	0.0863	-105.4359	0.9261
Phosphate buffer, Yeast extract, Potassium nitrate*Sodium chloride, Potassium nitrate*Yeast extract, Phosphate buffer*Bis-Tris, Bis-Tris*Yeast extract	6	0.841	0.0851	-105.4316	0.9304
Yeast extract, Potassium nitrate*Sodium chloride, Potassium nitrate*Yeast extract, Phosphate buffer*Bis-Tris, Phosphate buffer*Yeast extract, Bis-Tris*Yeast extract	6	0.8406	0.0852	-105.269	1.093
Phosphate buffer, Yeast extract, Potassium nitrate*Sodium chloride, Sodium chloride*Phosphate buffer, Phosphate buffer*Bis-Tris, Bis-Tris*Yeast extract	6	0.8401	0.0854	-105.111	1.251
Phosphate buffer, Yeast extract, Potassium nitrate*Sodium chloride, Phosphate buffer*Bis-Tris, Bis-Tris*Yeast extract	5	0.8307	0.087	-104.6427	1.7193
Phosphate buffer, Yeast extract, Potassium nitrate*Sodium chloride, Potassium nitrate*Phosphate buffer, Phosphate buffer*Bis-Tris, Phosphate buffer*Yeast extract	6	0.8386	0.0858	-104.5895	1.7725
Phosphate buffer, Yeast extract, Ammonium sulphate*Yeast extract, Potassium nitrate*Sodium chloride, Phosphate buffer*Bis-Tris, Phosphate buffer*Yeast extract	6	0.8384	0.0858	-104.5265	1.8355
Sodium chloride, Phosphate buffer, Yeast extract, Potassium nitrate*Sodium chloride, Phosphate buffer*Bis-Tris, Phosphate buffer*Yeast extract	6	0.8383	0.0858	-104.4707	1.8913
Ammonium chloride, Phosphate buffer, Yeast extract, Potassium nitrate*Sodium chloride, Phosphate buffer*Bis-Tris, Bis-Tris*Yeast extract	6	0.8382	0.0859	-104.4308	1.9312
Phosphate buffer, Yeast extract, Ammonium chloride*Sodium carbonate, Potassium nitrate*Sodium chloride, Phosphate buffer*Bis-Tris, Bis-Tris*Yeast extract	6	0.838	0.0859	-104.3734	1.9886
Phosphate buffer, Yeast extract, Potassium nitrate*Sodium chloride, Sodium carbonate*Phosphate buffer, Phosphate buffer*Bis-Tris, Phosphate buffer*Yeast extract	6	0.838	0.0859	-104.3707	1.9913

Table S8: Measures of fit for 15 stepwise regression models of the results of the second DoE iteration.
All possible linear regression models were fit to the data, with Heredity Restriction and a maximum of 6 terms per model. The goodness-of-fit of each of the resulting 9,531,039 models was assessed using the Akaike Information Criterion (AICc). For each candidate model (i), the Kullback-Leibler distance from the optimum model (*i.e.* the model with the smallest AICc) was calculated as

$\Delta_i = AICc_i - AICc_{min}$. Models were selected for further interrogation when Δ_i was less than 2.0 (Burnham *et al*., 2010). The 15 selected models are listed in the table in ascending order of Δ_i.
