# Supplementary material for: A design of experiments approach for the rapid formulation of a chemically defined medium for metabolic profiling of industrially important microbes
Source: PLoS One. 2019 Jun 12;14(6):e0218208. doi: 10.1371/journal.pone.0218208 (PMC6561596; doi:10.1371/journal.pone.0218208)
Supplement: S1 Fig — G. thermoglucosidans cultures were grown in 250 ml flask format in mDM supplemented with 10 g L-1 of either (A) hexose monosaccharides; (B) pentose monosaccharides; (C) disaccharides and (D) sugar alcohols, alpha-keto acid and dicarboxylic acid. All graphs show growth curves for G. thermoglucosidans cultures grown in mLB and mDM with no additional carbon source for comparison. In all cases, cultures were inoculated to an initial OD600 of 0.04 using starter cultures prepared by resuspending G. thermoglucosidans biomass taken from a confluent agar plate in 5 g L-1 yeast extract solution. Cultures were incubated at 60 °C, with shaking at 220 rpm. Points represent the mean change in OD600 of n = 3 biological replicates. Standard deviation error bars are shown, unless hidden by the points. The lines represent fits of the data using a one-site specific binding with Hill slope equation. (PDF) [file pone.0218208.s009.pdf]

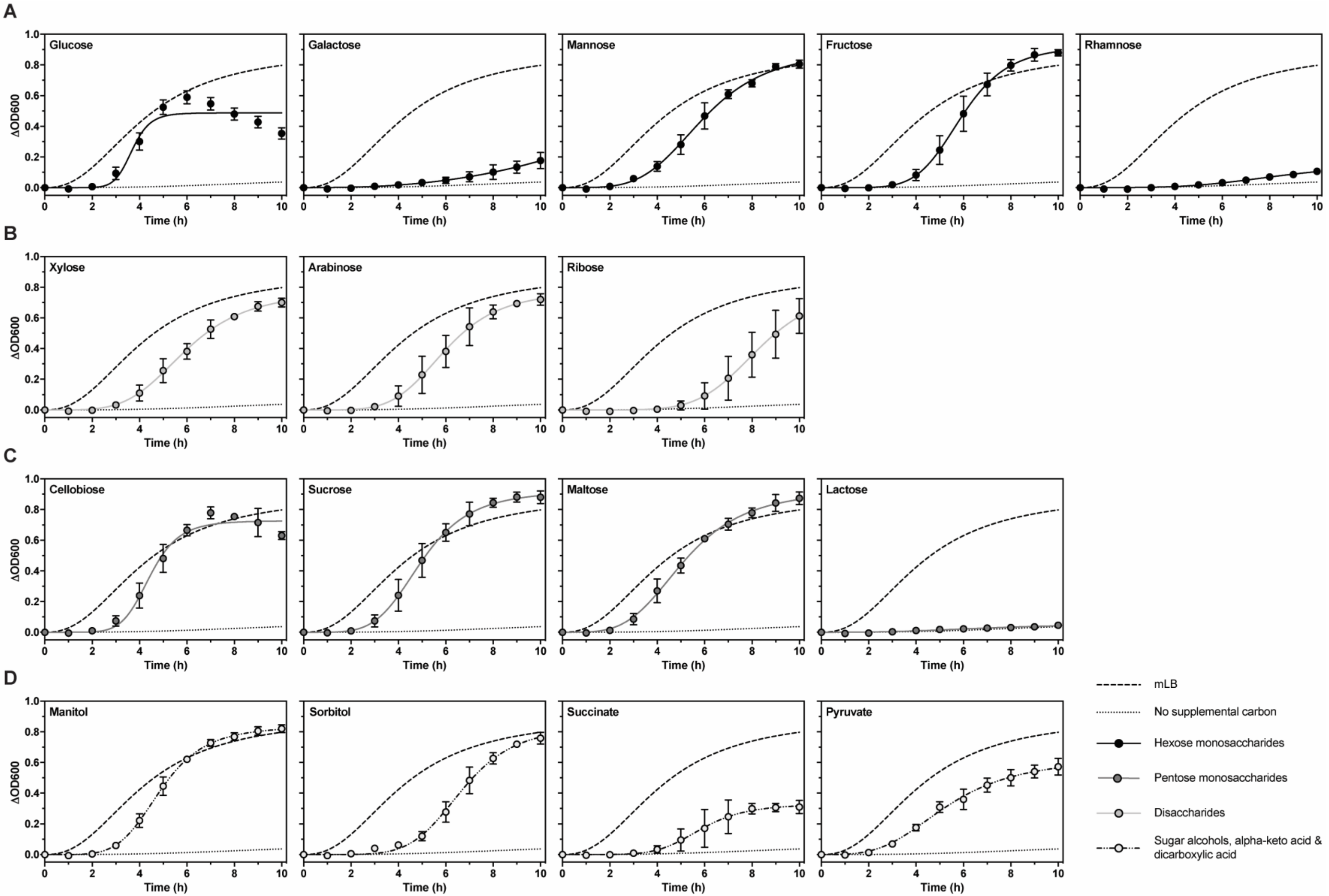
A
B
C
D
Glucose
Galactose
Mannose
Fructose
Rhamnose
Xylose
Arabinose
Ribose
Cellobiose
Sucrose
Maltose
Lactose
Manitol
Sorbitol
Succinate
Pyruvate
ΔOD600
Time (h)
mLB
No supplemental carbon
Hexose monosaccharides
Pentose monosaccharides
Disaccharides
Sugar alcohols, alpha-keto acid & dicarboxylic acid

**Figure S1: Growth of *G. thermoglucosidans* in the defined medium mDM supplemented with various carbon sources.**
*G. thermoglucosidans* cultures were grown in 250 ml flask format in mDM supplemented with 10 g $l^{-1}$ of the relevant carbon source. All graphs show growth curves for *G. thermoglucosidans* cultures grown in mLB and mDM with no additional carbon source for comparison. In all cases, cultures were inoculated to an initial OD600 of 0.04 using starter cultures prepared by resuspending *G. thermoglucosidans* biomass taken from a confluent agar plate in 5 g $l^{-1}$ yeast extract solution. Cultures were incubated at 60 °C, with shaking at 220 rpm. Points represent the mean change in OD600 of n = 3 biological replicates. Standard deviation error bars are shown, unless hidden by the points. The lines represent fits of the data using a one-site specific binding with Hill slope equation.
